# Serum HER 2 Extracellular Domain Level Is Correlated with Tissue HER 2 Status in Metastatic Gastric or Gastro-Oesophageal Junction Adenocarcinoma

**DOI:** 10.1371/journal.pone.0063458

**Published:** 2013-05-14

**Authors:** Shu-Qin Dai, Xin An, Fang Wang, Qiong Shao, Yong-Chang Chen, Ya-Nan Kong, Cui Chen, Cong Li, Hui-Yan Luo, Ying Liang, Feng-Hua Wang, Rui-Hua Xu, Yu- Hong Li

**Affiliations:** 1 State Key Laboratory of Oncology in South China, Guangzhou, China; 2 Department of Medical Examination, Sun Yat-sen University Cancer Centre, Guangzhou, China; 3 Department of Medical Oncology, Sun Yat-sen University Cancer Centre, Guangzhou, China; 4 Department of Molecular Pathology, Sun Yat-sen University Cancer Centre, Guangzhou, China; 5 Department of Breast Oncology, Sun Yat-sen University Cancer Centre, Guangzhou, China; Health Canada, Canada

## Abstract

**Background:**

To explore the association between serum human epidermal growth factor receptor 2 (HER 2) extracellular domain (ECD) levels and tissue HER 2 status in metastatic gastric cancer.

**Patients and Methods:**

HER 2 status was retrospectively analyzed in 219 advanced gastric or gastroesophageal junction (GEJ) patients. Serum HER 2 ECD was measured by chemiluminescent assay and tissue HER 2 was assessed by fluorescent in situ hybridisation (FISH) and immunohistochemistry (IHC) assay.

**Results:**

Significant associations were found between serum HER 2 ECD levels and tissue HER 2 status. Twenty-four patients had HER 2 ECD levels >16.35 ng/mL, which has a sensitivity of 51.4% and a specificity of 97.3% to predict tissue HER 2 status. When the cut-off value was increased to 22 ng/mL, then all 12 patients with serum HER 2 ECD levels>22 ng/mL were tissue HER 2 positive, corresponding to a specificity of 100% and a sensitivity of 32.4%. High serum HER 2 ECD levels were strongly associated with the intestinal histological type (Lauren’s classification), liver metastasis, multiple metastasis (>2) and increased LDH levels, but not with overall survival.

**Conclusions:**

The high specificity of the serum HER 2 ECD assay in predicting tissue HER 2 status suggests its potential as a surrogate marker of the HER 2 status in gastric cancer.

## Introduction

Human epidermal growth factor receptor 2 (HER 2) is a 185- kDa transmembrane protein encoded by HER2/neu or the c-erbB-2 proto-oncogene on chromosome 17q21. It is a member of the HER family of transmembrane receptors that are involved in regulating of many different cellular processes, including proliferation, differentiation, migration, and survival [1]. HER 2 overexpression occurs in 7–34% of gastric and gastroesophageal junction (GEJ) adenocarcinomas using different scoring methods or assays [2], [3]. Increasing evidence suggests that HER 2 is an important biomarker and a novel therapeutic target in gastric cancer and GEJ adenocarcinoma. [4]. The results of a phase III ToGA trial demonstrated a survival benefit with the HER 2-targeting monoclonal antibody trastuzumab plus chemotherapy (capecitabine or 5-fluorouracil and cisplatin) in patients with HER 2-positive advanced gastric or GEJ cancer [5]. On the basis of these trial results, it is now recommended that patients with advanced gastric and GEJ adenocarcinoma should be tested for tissue HER 2 status by immunohistochemistry (IHC) and fluorescence in situ hybridisation (FISH) in order to guide anti-HER 2 therapy. However, both assays have their own limitations: 1) Each technique requires a high-quality tissue sample, which sometimes may not be available; (2) There is a lack of “real-time” monitoring during anti-HER 2 therapy; (3) Discordance between IHC and the FISH results may occur due to interlaboratory variability, tumour heterogeneity, antigen loss during tissue storage and processing, non-standardized procedures, subjective observations, and discrepancies of HER2 protein expression and gene amplification. The American Society of Clinical Oncology – College of American Pathologists (ASCO-CAP) guidelines warn that the current HER 2 testing methods may be inaccurate in up to 20% of cases in breast cancer [6]. Because gastric cancer exhibits a high incidence of tumour heterogeneity in up to 30% of HER 2-positive cases [7], the inaccuracy rate may be even higher. Therefore, the search for an easy, accurate and reliable complementary method for HER 2 testing continues.

The HER 2 protein has three domains: a 105-kDa extracellular domain (ECD), a short transmembrane region, and an intracellular tyrosine kinase domain. The ECD of HER 2 can be cleaved from the surface of cancer cells and released into the serum, known as ECD shedding, a process that can be measured with enzyme-linked immunosorbent assays (ELLSAs) without any significant cross-reactivity with other members of the HER receptor family. [8]. In contrast to tumour tissue, serum samples can be easily and repeatedly obtained. Moreover, serum HER 2 levels can be easily measured and quantified with an automated platform, thus generating considerable interest as a supplement to tissue-based HER 2 testing. Elevated serum HER 2 ECD levels in breast cancer patients have been documented in many studies, and in most cases, serum HER 2 ECD levels are in good concordance with primary breast tumour HER2 status [9], [10], [11]. However, the prevalence and clinical application of serum HER 2 ECD in gastric cancer have not been explored. Here, we performed a retrospective analysis in a large series of metastatic gastric or GEJ adenocarcinoma patients to evaluate the correlation between serum HER 2 ECD levels and tissue HER 2 status determined by IHC and FISH,their relationship with clinical–pathological parameters and the impact on overall survival (OS).

## Materials and Methods

The study was approved by the Research Ethics Committee of the Sun Yat-sen University Cancer Centre. A total of 219 unselected cases of histologically confirmed, inoperable, locally advanced, recurrent, or metastatic gastric or GEJ adenocarcinoma that were referred to SunYat-Sen University Cancer Centre between October 2004 and March 2012 were enrolled. All patients provided written informed consent according to the institutional guidelines. Serum samples were collected at the time of metastatic disease diagnosis and were stored at −80°C. HER 2 status in primary tumour was available for all patients (219 patients underwent HER 2 FISH tests, and 170 patients had HER 2 IHC tests). Clinicopathological data, including age, performance status, tumour grade, metastatic sites, tumour markers, systemic chemotherapy regimens, and OS were collected.

### Serum HER2 ECD Assays

Serum HER 2 ECD was measured by a 2-site chemiluminescence’s sandwich immunoassay using an ADVIA Centaur System (Siemens Healthcare Diagnostics, Deerfield, IL, USA) with a detection range of 0.5–350 ng/mL. Measurements were performed strictly according to the manufacturer’s instructions and quality control was ensured.

### Tissue HER 2 Assessment by IHC and FISH

HER 2 amplification was assessed using a Spectrum Green fluorophore-labelled a-satellite DNA probe for chromosome 17 (Chr17) and a Spectrum Orange fluorophore-labelled DNA probe for the HER 2 gene locus (Vysis, Abbott Laboratories, IL) following the manufacturer’s recommended protocol, as we previously described [12]. FISH signals for each locus-specific FISH probe were assessed under an Olympus BX51 TRF microscope (Olympus, Japan) equipped with a triple-pass filter (DAPI/Green/Orange Vysis). The results were reported as the ratio between the average copy number of the HER 2/neu gene and that of the chromosome 17 centromere for 100 neoplastic nuclei. Amplification was defined as a HER2/CEP17 ratio ≥2 or when an HER 2 signal cluster was observed [13].

HER2 IHC was performed on formalin-fixed, paraffin-embedded tissue using the Ventana anti-HER 2/neu (4B5) Rabbit Monoclonal Primary Antibody kit (Ventana/Roche Tissue Diagnostics) following the manufacturer’s instructions. HER 2 immunoreactivity was evaluated by an experienced pathologist according to the scoring system proposed by Hofmann et al [13] and Rüschoff et al [14]. For resection samples, the following indications were used: 0, no staining or membranous reactivity in <10% of tumour cells; 1+, weak, barely perceptible membranous reactivity in >10% of tumour cells; 2+, complete or basolateral membranous reactivity, either non-uniform or weak, in at least 10% of cells; and 3+, strong complete or basolateral membranous reactivity of strong intensity in ≥10% of cells. The same patterns were considered for tumour biopsy specimens, but the percentage of tumour cells was not considered. Pathologists who were blinded to patients’ clinical characteristics and all molecular variables independently performed FISH and IHC analyses.

### Statistical Analyses

Sample size was estimated according to our preliminary study results showing 15% of 111 patients had an increased serum HER 2 ECD level. As a result, an estimated sample size of 196 would have been required to have at least 90% power at α = 0.05 to detect the difference between the two HER 2 assays with a margin of error of 0.05. Considering a 10% dropout rate, we increased the number of patients to 219. All statistical analyses were conducted using the SPSS 15.0 statistical software program (Chicago, IL, USA). Receiver operating characteristic (ROC) curve analysis was performed to determine an optimal HER 2 ECD value to predict the tissue HER 2 status. Serum HER 2 ECD levels among different levels of HER 2 gene amplification and protein expression groups were accomplished by Kruskal-Wallis H test. The correlation between serum HER 2 ECD levels and tissue HER 2 status as well as the association between serum HER 2 ECD levels and clinical–pathological features were analyzed with the chi-square tests. Survival curves were plotted using the Kaplan–Meier method, and significant differences between these curves were determined using the log-rank tests. Results were considered significant when P-values were less than 0.05.

## Results

### Patient Characteristics

The median age of the 219 patients was 53 years old (range, 27 to 77 years); 135 (61.6%) patients were male, and 84 (38.4%) were female. Overall, 158 patients had gastric cancer, and 61 patients had GEJ adenocarcinoma. A total of 51 (23.3%) patients had previously undergone curative resection. Lymph nodes were the most common site of metastatic disease (119, 54.3%), followed by the peritoneum (109, 49.8%), liver (55, 25.1%) and lung (19, 8.7%). The majority of patients (199, 90.9%) had received first-line chemotherapy: 69 (34.7%) patients were treated with cisplatin/fluoropyrimidine-based therapy; 60 (30.2%) patients received taxane-based chemotherapy; 54 (27.1%) patients received oxaliplatin-based chemotherapy; and 16 (8.0%) patients received irinotecan-based chemotherapy. Nearly half patients (94, 42.9%) received second-line chemotherapy: 43 (45.7%) patients received taxane-based chemotherapy; 25 patients (26.6%) received oxaliplatin-based chemotherapy; 16 patients (17.0%) received irinotecan-based chemotherapy; and 10 patients (10.6%) received other chemotherapy. Altogether, 17 (7.8%) patients received anti-HER 2 therapy (including trastuzumab or lapatinib).

### Association between Serum HER 2 ECD and Tissue HER 2 Status

**Figure 1:**
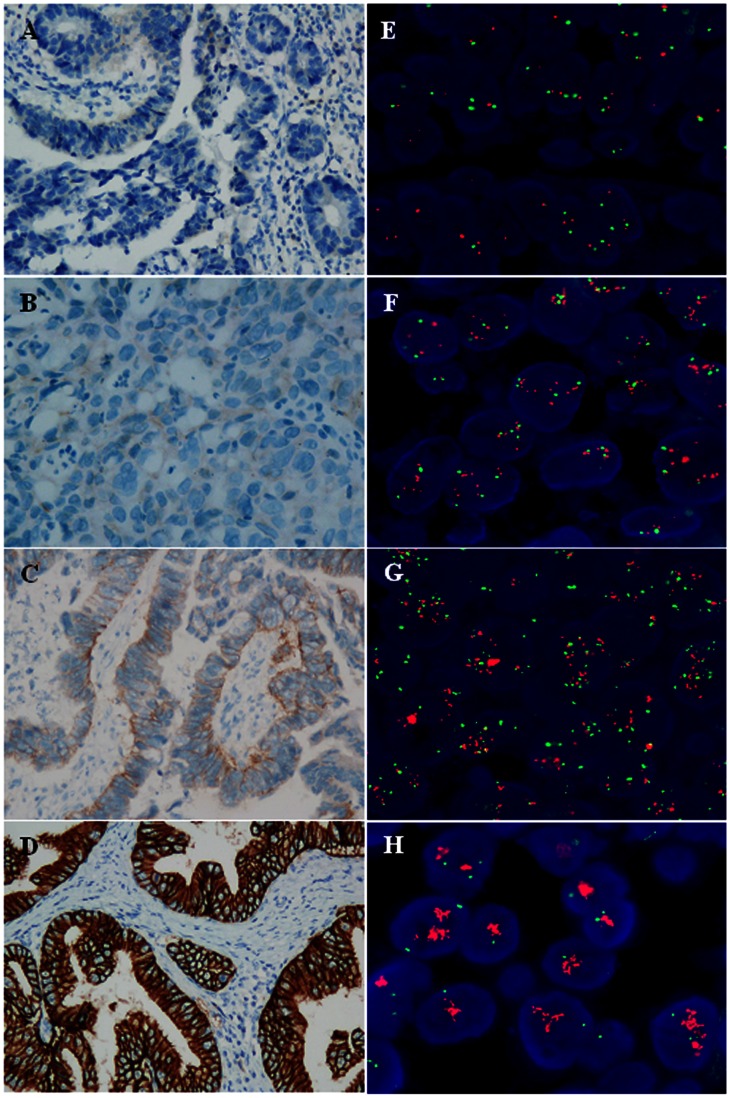
HER 2 protein expression and gene amplification in gastric cancer tissue. A–D show HER 2 protein expression detected by IHC (original magnification ×200). A, HER 2 IHC score of 0; B, 1+; C, 2+; D, 3+. E–H show HER 2 gene amplification evaluated by FISH (original magnification ×1000). E, no amplification; F, low level of amplification; G, moderate level of amplification; H, high level of amplification (HER 2 signal cluster).

Thirty-five (16.0%) patients showed HER 2 gene amplification by FISH analysis, including 30 patients with a high level of amplification (gene-to-chromosome ratio >10 or a HER 2 cluster pattern), 2 with a moderate level of amplification (gene-to-chromosome ratio ranging between 5.0 and 10.0), and 3 with a low level of amplification (gene-to-chromosome ratio ranging between 2.0 and 5.0). HER 2 IHC data were available for 170 patients: 22 (12.9%) showed a strong immunopositive (3+) reaction, 6 (3.5%) had a moderate immunopositive (2+) reaction and 142 (83.5%) exhibited weak or no immunohistochemical staining (0 or 1+, respectively). Tissue HER 2 status was positive (3+ by IHC and 2+ by IHC with FISH amplification) in 37 patients (16.9%) and negative in 182 patients (83.1%) (Figure 1).

**Figure 2:**
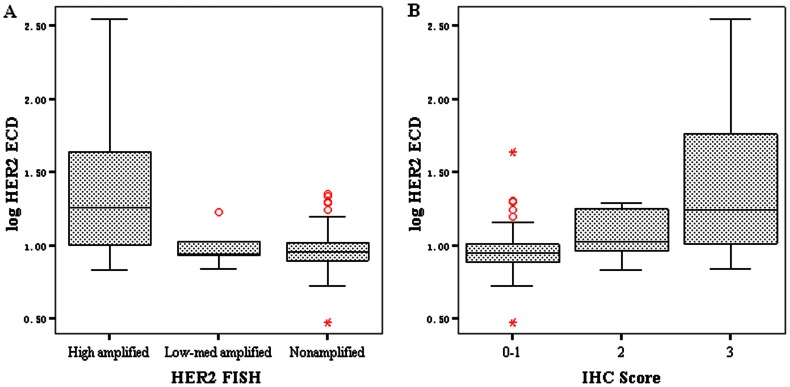
Serum HER 2 ECD levels stratified by different levels of tissue HER 2 amplification or expression. The median HER 2 ECD level was significantly higher in patients with high levels of HER 2 amplification than in patients with low to moderate levels of amplification or no amplification (A). The median HER 2 ECD level was significantly higher in patients with HER 2 IHC 3+ than in patients with HER 0–2+ (B).

The median serum ECD level in all 219 patients was 9.3 ng/mL (range 3.0 to >350) in all 219 patients. The median HER2 ECD level was significantly higher in patients with high levels of HER 2 amplification compared to patients with low to moderate levels of amplification or no amplification, 18.2 (6.8 to >350) ng/mL vs. 8.7(6.9–16.9) ng/mL vs. 9.0 (3.0–19.8) ng/mL, P<0.001. The median HER 2 ECD level was significantly higher in patients with HER 2 IHC 3+ than in patients with HER 2 IHC 2+ or HER2 IHC 0–1+, 17.6 (6.9 to >350) ng/mL vs. 10.6 (6.8–19.4) ng/mL vs. 8.9 (3.0–43.4) ng/mL, P<0.001 (Figure 2).

**Figure 3:**
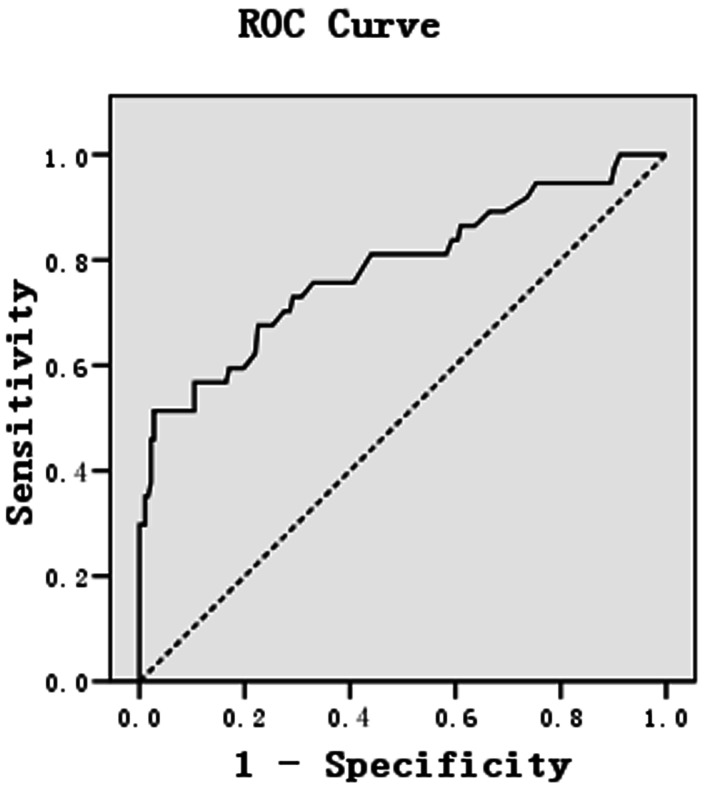
ROC curve for selection of the best cut-off value of serum HER 2 ECD to predict tissue HER 2 status. A cut-off of 16.35 ng/mL has a sensitivity of 51.4% and a specificity of 97.3% in discriminating HER 2-positive and HER 2-negative tumours.

To identify an optimal cut-off value of serum HER 2 ECD, we first used 15 ng/mL as criteria recommended by the FDA for breast cancer. Overall, 26 of the 219 (11.9%) patients had HER 2 ECD levels >15 ng/mL and 193 (88.1%) patients had HER 2 ECD levels <15 ng/mL, corresponding to 96.2% specificity and 51.4% sensitivity in predicting tissue HER 2 status. A ROC-analysis suggested that the cut-off of 16.35 ng/mL (24 patients had HER 2 ECD levels >16.35 ng/mL) could produce the same sensitivity (51.4%) and greater specificity (97.3%) (Figure 3). When the cut-off value was further increased to 22 ng/mL, all 12 patients with serum HER 2 ECD levels>22 ng/mL were HER 2 positive in primary tumour, corresponding to a specificity of 100% and a sensitivity of 32.4% (Table 1).

**Table 1:** Association between serum HER 2 ECD levels and tissue HER 2 status (with different cut-off points).

	HER 2 status		Sensitivity (%)	Specifity (%)
	Positive	Negative	95% CI	95%CI
**Cut-off point A**			51.4(32.9–69.0)	96.2(85.58–97.19)
(15 ng/mL)				
Elevated HER 2 ECD	19	7		
Normal HER 2 ECD	18	175		
*P* value (χ2 test)	<0.001			
**Cut-off point B**			51.4(32.9–69.0)	97.3(88.33–98.38)
(16.35 ng/mL)				
Elevated HER 2 ECD	19	5		
Normal HER 2 ECD	18	177		
*P* value (χ2 test)	<0.001			
**Cuto-ff point C**			32.4(17.5–51.0)	100(96.31–100)
(22 ng/mL)				
Elevated HER 2 ECD	12	0		
Normal HER 2 ECD	25	182		
*P* value (χ2 test)	<0.001			

### Relationship between Serum HER 2 ECD Levels and Clinicopathological Variables

High serum HER 2 ECD levels were strongly associated with the intestinal histological type (according to Lauren’s classification) (P = 0.003), liver metastasis (P<0.001), multiple metastases (>2) (P = 0.012) and an increased LDH level (P<0.001). High serum HER 2 ECD levels were more common in GEJ primary adenocarcinoma, however, the difference did not reach statistical significance. There was no association between serum HER2 ECD levels and age, gender, performance status, or metastatic sites (lung, peritoneum) (Table 2).

**Table 2:** Relationships between serum HER2 ECD levels and clinicopathological variables.

Variable	Elevated ECD	Normal ECD	P Value
	*n = 24*	*n = 195*	
Median age	52±10(27–77)	56±10(32–70)	0.15
Gender			0.383
Male	17(12.5%)	119 (87.5%)	
Female	7 (91.6%)	76 (8.4%)	
ECOG performance status			0.792
0–1	20 (11.6%)	152 (88.4%)	
≥2	4(8.5%)	43(91.5%)	
Primary tumour site			0.052
Stomach	13(8.2%)	145 (91.8%)	
Gastro-oesophageal junction	11(18.0%)	50 (82.0%)	
Type of gastric cancer			0.003
Intestinal	22 (22.9%)	74 (77.1%)	
Diffuse or Mixed	2 (1.6%)	121(98.4%)	
Number of metastasis			0.012
1–2	24(12.3%)	171 (87.7%)	
>2	8 (33.3%)	16 (66.7%)	
Peritoneum metastasis			0.05
Yes	7(6.4%)	102(93.6%)	
No	17(15.5%)	93(84.5%)	
Liver metastasis			<0.001
Yes	18 (31.0%)	40 (69.0%)	
No	6 (3.7%)	155 (96.3%)	
Lung metastasis			0.446
Yes	3(15.8%)	16(84.2%)	
No	21(10.5%)	179(89.5%)	
Baseline LDH (U/L)			<0.001
<245	13(7.4%)	175(92.6%)	
** >**245	13(41.9%)	18(58.1%)	

### Survival Analysis

**Figure 4:**
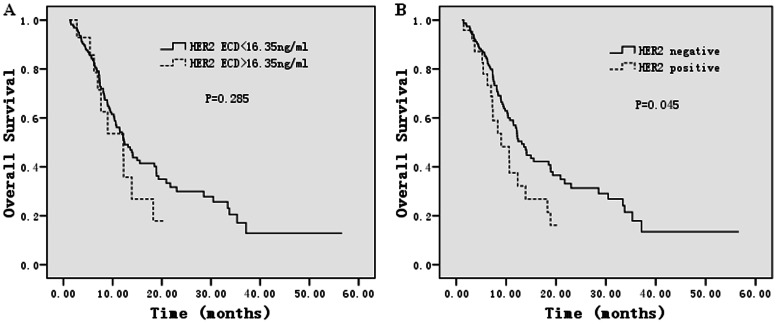
Kaplan–Meier estimates for overall survival according to serum HER 2 ECD levels and tissue HER 2 status. Serum HER 2 ECD levels showed no association with overall survival. Whereas positive tissue HER 2 status was significantly associated with worse overall survival.

To explore the prognostic role of HER 2 ECD, we excluded 20 patients who did not undergo chemotherapy after diagnosis of metastasis, and the 17 patients who were treated with anti-HER 2 therapy. The median follow-up time was 20 months and 115 patients died. The survival analyses demonstrated that serum HER 2 ECD levels were not associated with OS. The median OS for patients with HER 2 ECD >16.35 ng/mL was 11.2 months (95% confidence interval 5.0 to 19.3 months), while the median OS for patients with HER 2 ECD <16.35 ng/mL was 12.4 months (95% confidence interval 10.1 to 14.7 months), P = 0.285 (Figure 4A). However, Univariate and multivariate analysis showed that positive tumour HER 2 status was an independent risk factor for OS, HR = 1.371(1.058–2.191), P = 0.042. HER 2 tissue status was significantly associated with OS. The median OS for patients with positive HER 2 tissue status was 9.0 months (95% confidence interval 4.6 to 13.4 months), while the median OS for patients with negative tissue HER 2 status was 13.1 months (95% confidence interval 11.3 to 15.0 months), P = 0.045 (Figure 4B ).

## Discussion

For the first time, the present study retrospectively detected serum HER 2 ECD levels in a large series of patients with metastatic gastric or GEJ adenocarcinoma. Many studies have measured serum HER 2 ECD levels in breast cancer patients and showed that HER 2 ECD was increased in 9% to 22.9% of patients in early stage disease [9], [15], and in 22% to 73% of patients in advanced stage disease [11], [16], [17]. In the current study of gastric cancer patients, when a cut-off value of 15 ng/mL was used, as for breast cancer, 11.9% of patients showed increased serum HER 2 ECD levels. When a cut-off value of 16.35 ng/mL (produced by the ROC curve) was used, 11.0% of patients met the cut-off for increased serum HER 2 ECD levels. Therefore, the prevalence of increased HER 2 ECD appears to be lower in gastric cancer patients compared with breast cancer.

One of the potential applications of serum HER 2 ECD detection is to consider it to be a complementary method to predict tissue HER 2 status. In breast cancer, a variety of studies have demonstrated good concordance between serum HER 2 ECD levels and tissue HER 2 tests [9], [11], [18], [19]. Statistical analysis of our results also showed a strong correlation of serum HER 2 ECD concentration with tissue HER 2 status. Serum HER 2 ECD levels were significantly higher in patients with both HER 2 gene amplification and/or protein overexpression than in those without HER 2 gene amplification and/or protein overexpression. ROC analysis showed 97.3% specificity and 51.4% sensitivity of serum HER 2 ECD for predicting tissue HER 2 positivity when a cut-off of 16.35 ng/mL was applied. When the cut-off value was further increased to 22 ng/mL, all 12 patients with serum HER 2 ECD levels >22 ng/mL had HER 2 positive in primary tumour samples, corresponding to a specificity of 100% and a sensitivity of 32.4%. These results suggest that the serum HER 2 ECD assay exhibits high specificity but low sensitivity. However, it is still rational to consider it to be a surrogate marker of HER 2 status in metastatic gastric cancer patients. As previously discussed, IHC and FISH have been shown to have some limitations. Serum HER 2 ECD represents a non-invasive and quantifiable biomarker that may supplement existing tissue-based HER 2 testing. For instance, it may provide useful HER 2 status information for metastatic gastric cancer patients who lack tissue samples for IHC and FISH testing. In breast cancer, recent studies showed that the dynamic change of serum HER 2 ECD levels could be used for predicting a patient’s response to trastuzumab or for early detection of relapse or progression [20], [21]. In gastric cancer, a small phase II study conducted by Grávalos et al detected HER 2 ECD levels in 22 HER 2-positive gastric cancer patients and showed that higher baseline HER 2 ECD levels were associated with better outcome in terms of disease control and survival [22]. Further studies are warranted to evaluate its predictive role of anti- HER 2 treatment or early metastatic detection in gastric cancer.

A number of breast cancer studies demonstrated that elevated serum HER 2 ECD is associated with high grade tumour, larger tumour burden, lymph node involvement, higher recurrence rate and higher mortality, thus, it was associated with a poorer prognosis [9], [22], [23]. In our study, elevated levels of HER 2 ECD were also strongly associated with liver metastasis and high tumour load (multiple metastases and increased LDH levels). In addition, our results showed elevated levels of HER 2 ECD almost exclusively in patients with the intestinal phenotype according to Lauren’s classification (except one patient with the diffuse type). The survival analysis showed that positive tissue HER2 status was strongly associated with worse survival rate, which is in consistent with the most recent meta-analysis [24]. Although patients with elevated HER 2 ECD who received cytotoxic chemotherapy tended to have a shorter overall survival, this trend did not reach statistical significance, possibly due to the limited number of patients with elevated serum levels of HER 2 ECD.

In conclusion, the current study demonstrated a significant correlation between increased serum HER 2 ECD levels and positive tissue HER 2 status as assessed by IHC and FISH in a large series of patients with metastatic gastric cancer or GEJ adenocarcinoma. The serum HER 2 ECD assay shows high specificity, suggesting its potential as a surrogate marker of the HER2 status in metastatic gastric cancer patients. A perspective study is need to validate our findings, and dynamic monitoring of serum HER 2 ECD for predicting response to anti-HER2 therapy is worthy of further consideration.
